# Schwannoma of ascending colon treated by laparoscopic right hemicolectomy

**DOI:** 10.1186/1477-7819-10-81

**Published:** 2012-05-15

**Authors:** Hun Jin Kim, Chang Hyung Kim, Sang Woo Lim, Jung Wook Huh, Young Jin Kim, Hyeong Rok Kim

**Affiliations:** 1 Department of Surgery, Chonnam National University Hwasun Hospital and Medical School, Gwangju, Korea; 2 Department of Surgery, Chonnam National University Hwasun Hospital and Medical School, 160 Ilsimri, Hwasun-eup, Hwasun-gun, Jeonnam 519-809, Korea

**Keywords:** Schwannoma, Ascending colon, Immunohistochemistry

## Abstract

Schwannomas of the colon are rare and are difficult to diagnose preoperatively, since they often defy endoscopic and radiographic detection. Immunohistochemical stains are useful postoperatively to confirm this tumor, but more reliable diagnostic techniques (such as colonoscopic biopsy with immunohistochemistry) have emerged to enhance preoperative diagnostic accuracy. Here we report an instance of schwannoma arising in the ascending colon, where immunohistochemical staining of a preoperative biopsy facilitated diagnosis. After laparoscopic resection, histologic examination was confirmatory.

## Background

Schwannomas of the gastrointestinal tract are relatively uncommon and rarely involve the large intestine [1-3]. Although considered benign, they may recur locally (if incompletely excised), and malignant transformation is occasionally observed [4,5]. Radical surgery is, therefore, the accepted standard of treatment.

While accurate diagnosis prior to surgical intervention can aid in therapeutic planning, limitations of conventional imaging and lack of sufficient biopsy material usually make this difficult. Recent improvements in colonoscopic techniques have led to use of endoscopic biopsy and immunohistochemistry in combination as a more reliable method for accurate preoperative assessment. We report here an instance of schwannoma of the ascending colon, confirmed by immunohistochemistry, for which laparoscopic right hemicolectomy was performed.

## Case report

**Figure 1:**
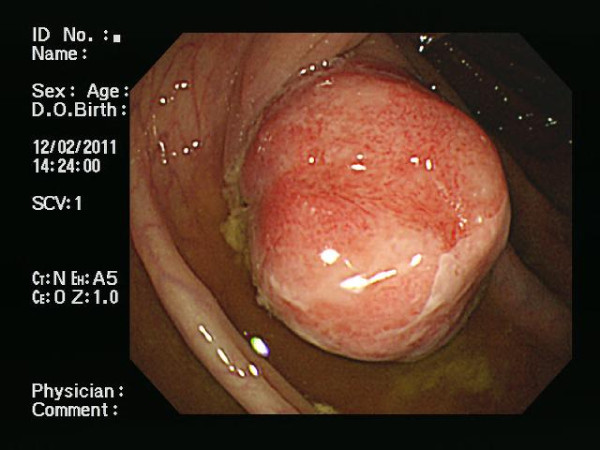
Semipedunculated polypoid lesion (2 cm) of the ascending colon by colonoscopy: note coating of whitish exudate.

A 61-year-old male was referred to his local hospital for a routine health examination. On screening colonoscopy, a polypoid lesion of the ascending colon was discovered (Figure 1). A subsequent biopsy showed only signs of chronic ulceration (fibrinoid necrosis and neutrophils), so he was transferred to our hospital for further evaluation and treatment.

**Figure 2:**
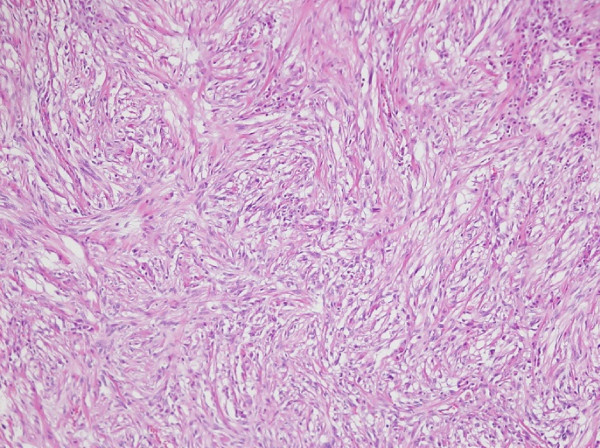
Microscopic view of mass showing spindle cell aggregate (10X, 100X).

**Figure 3:**
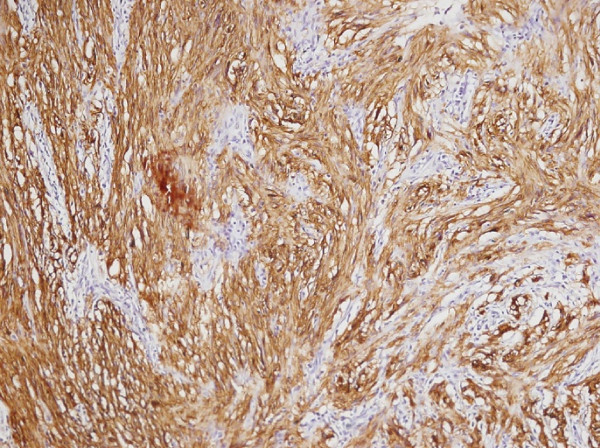
Strong immunoreactivity of tumor cells to S-100 protein.

The patient’s physical examination was noncontributory, but we obtained multiple colonic biopsies. The mass was composed of benign spindle cells (Figure 2) strongly positive for S-100 protein and negative for smooth muscle actin, CD117 and CD34 (Figure 3). This histochemical profile was compatible with schwannoma.

**Figure 4:**
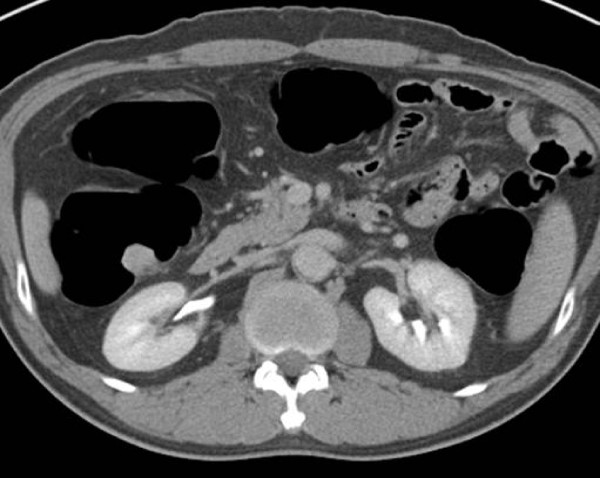
Homogeneous enhancement of mass on abdominopelvic CT.

**Figure 5:**
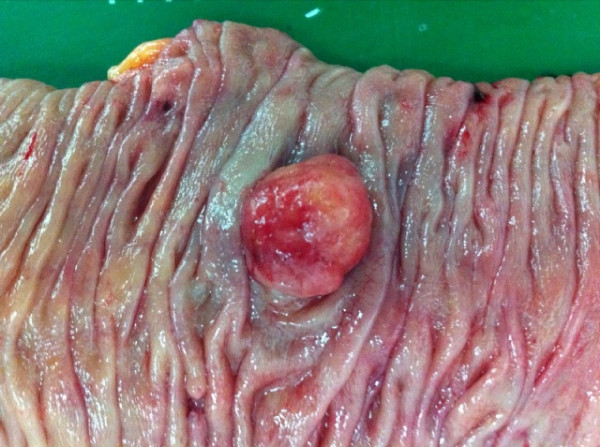
Gross findings: 1.7 cm × 1.8 cm well-circumscribed polypoid growth.

Abdominopelvic computed tomography (CT) indicated that the lesion of the ascending colon protruded intraluminally, exhibiting homogenous enhancement (Figure 4). A well- circumscribed, fungating mass (1.7 cm × 1.8 cm) was ultimately resected via laparoscopic-enabled right hemicolectomy with side-to-side ileocolic anastomosis (Figure 5). Histologic evaluation, including immunohistochemistry, confirmed the tumor as schwannoma. Postoperatively, the patient recovered and has done well with no recurrence.

## Discussion

Schwannomas are uncommon neoplasms arising from Schwann cells of the neural sheath [2,6]. They may occur anywhere in the body, but gastrointestinal sites are rare, especially the colon [1-3,7-12]. Depending on tumor size and location, schwannomas of the colon may occasionally produce symptoms, such as constipation, bleeding, abdominal pain or discomfort, and anal pain [2,10]. Intussuception is unusual [12]. Most patients, however, have no symptoms, because these are inherently slow-growing neoplasms [9,13]. In our patient, tumor discovery was incidental.

Preoperative diagnosis of schwannoma is difficult due to its tissue density and the tendency for ulceration [2]. Biopsies are often nondiagnostic, and information gained by other means is limited. Colonoscopy, abdominal ultrasound (US), abdominal CT and abdominal magnetic resonance imaging (MRI) may aid in evaluating the contours of colorectal schwannomas and their relationship with surrounding organs, as well as tumor multiplicity or metastasis. Levy, *et al*. [14] described gastrointestinal schwannomas as homogeneously attenuated and well-defined mural masses on CT, noting that they were indistinguishable from gastrointestinal stromal tumors. Low-attenuation hemorrhage, necrosis and internal degeneration constituted shared CT features. Attributes signifying benign vs malignant behavior were not addressed [15,16]. More recently, endoscopic ultrasonography (EUS) surpassed other imaging modalities in diagnostic accuracy but still will not differentiate a schwannoma from other gastrointestinal stromal tumors. EUS-guided fine needle aspiration or biopsy of submucosal tumors has been attempted [17-20].

Schwannoma of the colon usually is not diagnosable solely by routine histology. A newer, more reliable approach incorporates immunohistochemistry [2,7]. Characteristically, these tumors are composed of spindle cells (much like neurofibromas, gastrointestinal stromal tumors and leiomyomas) [7,21] that are 100% immunoreactive for S-100 protein. Cells of neurofibroma show less S-100 positivity (30% to 40%), while gastrointestinal stromal tumors are generally positive for CD117(c-kit) and CD34 (70%) and negative for S-100 protein. Leiomyomas similarly are devoid of S-100 protein, expressing smooth muscle actin and desmin instead [22,23].

Because the prognosis for schwannoma differs from other gastrointestinal stromal tumors, a correct diagnosis is critical. Schwannomas overall are considered benign, but they may recur locally if excision is incomplete and in rare instances are capable of malignant transformation [4,5]. Tozbikian *et al*. [5] found that gastric schwannomas on occasion could be aggressive, progressing rapidly and responding poorly to chemotherapy.

Standard treatment for schwannomas is complete surgical resection [4,24]. The role of radiotherapy or chemotherapy to date remains unclear [4,25]. Lymph node resection is not recommended, because the risk of malignant change is low [24,26]. A minimally invasive procedure (in our case, laparoscopic surgery) is acceptable for schwannoma of the colon and rectum [8,13,27]. Our patient resumed oral feeding on the second postoperative day and after an uneventful stay, was discharged on the sixth postoperative day. In clinical follow-up, he has been well and problem-free.

## Conclusion

In conclusion, schwannomas of the ascending colon are rare tumors dependent upon immunohistochemistry for definitive classification. Accurate preoperative diagnosis is essential for appropriate surgical management. While radical resection affords optimal outcomes, a laparoscopic approach may be used.

## Consent

Written informed consent was obtained from the patient for publication of this report and any accompanying images.

## Competing interests

The authors declare that they have no competing interests.

## Authors’ contributions

HJK wrote the main manuscript and HRK performed the operation, revised, the manuscript for important intellectual content, and gave the final, approval for the version to be submitted for publication. All authors read and approve the final manuscript.
